# Assessment of Biochemical, Inflammatory Biomarkers and Ultra-Processed Food Consumption in Children with Small Intestinal Bacterial Overgrowth: A Cross-Sectional Study

**DOI:** 10.3390/nu16152477

**Published:** 2024-07-31

**Authors:** Paulo César Trindade Costa, Davyson Barbosa Duarte, Thallyta Alanna Ferreira Viana das Neves, Rúbia Cartaxo Squizato de Moraes, Lydiane de Lima Tavares Toscano, Adélia da Costa Pereira de Arruda Neta, Vinicius José Baccin Martins, José Luiz de Brito Alves

**Affiliations:** 1 Department of Nutrition, Health Sciences Center, Federal University of Paraiba, João Pessoa 58051-900, PB, Brazil; paulocesarnutricionista@gmail.com (P.C.T.C.); davyson.duarte@ufpe.br (D.B.D.); thallytaviana@hotmail.com (T.A.F.V.d.N.); rubiacartaxo@gmail.com (R.C.S.d.M.); lyditavares@hotmail.com (L.d.L.T.T.); adeliacpereira@gmail.com (A.d.C.P.d.A.N.); 2 Department of Physiology and Pathology, Federal University of Paraíba, João Pessoa 58051-900, PB, Brazil; viniciusjbmartins@gmail.com

**Keywords:** children, small intestinal bacterial overgrowth, inflammation, biochemical parameters, food consumption

## Abstract

Objective: This study evaluated anthropometric, biochemical, and inflammatory biomarkers, as well as dietary intake in Brazilian children diagnosed with small intestinal bacterial overgrowth (SIBO) and compared them with their counterparts without SIBO. Methods: This was a cross-sectional study with 106 children aged 7 to 10 years. A glucose-hydrogen breath test was performed to diagnose small intestinal bacterial overgrowth (SIBO). Anthropometric and dietary characteristics were assessed. Blood samples were collected and serum biochemical parameters and cytokines were measured. Results: The occurrence of SIBO was 13.2%. Age, BMI, BMI/age WC, BFP, sex and biochemical markers were similar between SIBO-positive and SIBO-negative children (*p* > 0.05). High consumption of ultra-processed foods tended to be higher in SIBO-positive compared to SIBO-negative children (47.8 ± 8.2 vs. 42.6 ± 9.5, *p* = 0.06). Serum levels of IL-17 were higher in SIBO-positive than in SIBO-negative children [69.5 (5.4–125.7) vs. 53.4 (2.3–157.7), *p* = 0.03], while serum levels of IL-10 were lower in SIBO-positive than in SIBO-negative children [2.3 (0.6–7.2) vs. 5.7 (0.5–30.8), *p* = 0.04]. Finally, in a logistic regression adjusted for sex, BMI and age, consumption of ultra-processed foods (*p* = 0.03) and IL-6 levels (*p* = 0.003) were found to contribute to the occurrence of SIBO. Conclusion: this study identified for the first time an occurrence of 13% of SIBO in children living in the northeastern region of Brazil and showed that consumption of ultra-processed foods and serum levels of IL-6 may influence the occurrence of the SIBO in the pediatrics population.

## 1. Introduction

Impaired lifestyle has been associated with adverse health outcomes, impaired quality of life, and increased risk of gastrointestinal, metabolic, and inflammatory diseases in the pediatric population [1,2,3]. Hypercaloric diets, high consumption of ultra-processed foods, and low intake of fruits, natural vegetables, and fiber have been associated with obesity and related gastrointestinal, metabolic, and inflammatory diseases [4,5,6].

Early findings have shown that high consumption of ultra-processed foods may deleteriously affect the gut microbiota, disrupting intestinal homeostasis and promoting intestinal and systemic low-grade inflammation [7,8,9]. Excessive consumption of ultra-processed foods may be a risk factor for an overgrowth of certain bacteria and a decrease in beneficial bacteria in the gut [10].

Small intestinal bacterial overgrowth (SIBO) is a heterogeneous disorder characterized by excessive growth of selected microorganisms in the small intestine [11,12]. SIBO can disrupt host physiology and lead to gastrointestinal and non-gastrointestinal symptoms and complications [11,12]. The prevalence of SIBO in children has been studied in a wide range of clinical contexts and has been implicated as the cause of non-specific gastrointestinal symptoms such as chronic abdominal pain, bloating, diarrhea, and flatulence [11].

SIBO may be a risk factor for bacterial translocation, low-grade inflammation, and metabolic disorders in children [11]. However, the small number of studies has limited the understanding of the impact of SIBO on metabolic and inflammatory outcomes in children [13]. Therefore, this study evaluated biochemical and inflammatory biomarkers and dietary intake in children diagnosed with SIBO and compared them to their counterparts without SIBO.

## 2. Methods

### 2.1. Ethical Concerns

Following the Helsinki Declaration, this cross-sectional study was carried out. Approval was obtained from a Human Research Ethics Committee at the Federal University of Paraiba Health Sciences Center in João Pessoa, Brazil (Protocol number 4.676.103). All parents were informed about the study and provided written informed consent before data collection, following the guidelines outlined in Resolution 466/2012 of the National Health Council. The study received ethical approval on 28 April 2021. 

### 2.2. Members and Study Design

The study included children aged 7 to 10 years, of both genders, who were enrolled in public primary schools. Data collection and recruitment took place in schools from April 2022 to September 2023. Children were excluded if they had physical limitations preventing anthropometric measurements, psychological or behavioral disorders, were taking medications, or had any condition that could interfere with the analysis. A total of 106 children underwent hydrogen breath testing to diagnose small intestinal bacterial overgrowth (SIBO). Additionally, the children were assessed for anthropometry, body composition, and blood samples.

### 2.3. Hydrogen Breath Test Assessment

Before the breath test, both the children and their parents received specific instructions. They were required to fast for 12 h, avoiding milk or dairy products, high-fiber foods, sugary drinks, soft drinks, and industrial juices for 24 h before the test. Additionally, they were instructed not to use probiotics for 30 days leading up to the test. On the test day, the children were provided with an oral hygiene kit containing a toothbrush, toothpaste, and dental floss to ensure clean teeth. During the test, they blew slowly into a standardized device to establish the baseline breath hydrogen level. Following this, they ingested a standardized glucose solution (composed of 50 g of glucose and 250 mg of strawberry flavoring in 200 mL of water), and breath samples were collected before and after 15, 30, 60, 90, and 120 min after glucose intake [12]. Hydrogen breath testing was performed using the EasyH2-P00401 (Dynamed, São Paulo, Brazil). Breath samples were immediately analyzed for H2 using EasyH2 version 2.0 software and positivity was defined as an increase in H2 of ≥20 over the baseline [11]. Despite the limitations of the H2 test, it remains a useful tool in the diagnosis of pediatric SIBO due to its practical and non-invasive nature [11].

### 2.4. Anthropometry and Body Composition

In this study, we assessed anthropometry and body composition. Body weight was measured using an electronic scale (Omron^®^, model HBF-514C, São Paulo, Brazil), while height was determined using a stadiometer (alturaexata^®^, Belo Horizonte, Brazil). Nutritional status, based on body mass index for age (BMI/A) and sex, followed WHO standards and was determined using Anthro plus (version 1.0.4; WHO).

Waist circumference was measured using a flexible steel tape with a scale ranging from 0 to 200 cm and a precision of 0.1 mm (Sanny^®^, São Paulo, Brazil). Skinfold thickness was measured three times, always on the right side of the body, using a scientific adipometer with a precision of 0.1 mm (Sanny^®^). The average values from these measurements were then used to estimate body fat percentage. All assessments were conducted by a trained investigator.

### 2.5. Assessment of Food Consumption

To assess the food consumption of the children, we used a 2-day food record—a questionnaire administered on both a weekday and a weekend day. During the interviews carried out by trained nutritionists, a photographic album of Global Diet was used to clearly define portion sizes. Following the input of the 24-h data, a quality control procedure was carried out. Subsequently, the data were entered into the BRASIL NUTRI^®^ Software (Version 1), which was specifically designed for the 2017–2018 Family Budget Survey (known by its Portuguese acronym, POF). The foods were categorized into four groups based on the NOVA food classification: (1) unprocessed or minimally processed food, (2) processed culinary ingredients, (3) processed food, and (4) ultra-processed food. We calculated the average energy consumption (in kilocalories) over the two 24-h periods (R24h) based on the degree of food processing. By categorizing foods, we were able to calculate the energy intake specifically from unprocessed, processed, and ultra-processed foods. The sum of energy intake from groups 1, 2, 3, and 4 was used to obtain total caloric consumption. The energy of groups 1 and 2 were categorized together as unprocessed foods. Each relative proportion to total caloric consumption was determined as a percentage for unprocessed, processed, and ultra-processed foods.

### 2.6. Blood Samples and Biochemical and Cytokines Measurements

A qualified nurse collected blood samples from participants after a 12-h fasting period, during which they refrained from strenuous exercise in the preceding 24 h. Using an automated analyzer (Lab-Max 240, Labtest, Lagoa Santa, MG, Brazil) and standardized kits following the manufacturer’s instructions (Labtest, Lagoa Santa, MG, Brazil), the serum concentrations of various biomarkers were assessed. These included albumin, alanine transaminase (ALT), aspartate aminotransferase (AST), cholesterol, low-density lipoprotein cholesterol (LDL-c), high-density lipoprotein cholesterol (HDL-c), fasting glucose, glycated hemoglobin (HbA1c), and c-reactive protein (c-RP). Additionally, cytokines were measured using the Cytometric Bead Array (CBA) technique, as described in a previous study [2]. We employed Th1/Th2/Th17 CBA kits from Becton Dickinson Biosciences to quantify cytokines, including IL-2, IL-4, IL-6, IL-10, IL-17a, IFN-γ, and TNF-α. The fluorescence intensity of each complex corresponds to the cytokine concentration (measured in picograms per milliliter, pg/mL). For analysis, we utilized an Accuri C6 BD^®^ flow cytometer, and the CBA data were processed using FCAP 1.0.1 software.

### 2.7. Statistical Analysis

We evaluated data normality using the Kolmogorov-Smirnov test. Descriptive statistics were reported as either mean (standard deviation) or median (minimum–maximum). Group comparisons were conducted using Student’s *t*-test for parametric data and the Mann-Whitney test for non-parametric data. Additionally, logistic regression analysis was employed to further explore the relationships between small intestinal bacterial overgrowth (SIBO), consumption of ultra-processed foods, and serum cytokine levels. All statistical analyses were performed using GraphPad Prism^®^ (version 8.01), and significance was maintained at *p* < 0.05.

## 3. Results

The study found a SIBO occurrence of 13.2%. After classifying individuals into positive and negative SIBO, we performed comparative analyses on several variables. Age, BMI, BMI/A, Waist circumference (WC), body fat percentage (BFP) and gender showed no statistically significant differences (*p* > 0.05, Table 1). In addition, biochemical markers of lipid and liver function, glucose metabolism, albumin, and c-RP were similar between SIBO-positive and negative children (*p* > 0.05, Table 1).

The children tested as positive for SIBO had a similar consumption of calories, unprocessed or processed foods when compared to their SIBO-negative counterparts (*p* > 0.05, Table 2). However, children with a positive SIBO test had a higher consumption of ultra-processed foods, as indicated by a strong trend (*p* = 0.06, Table 2). Sweet biscuits, sugar added to food, industrialized chocolate milk products, salty cream cracker biscuits, industrialized juice powder, soft drinks, corn snacks, sausage, mortadella, and ham.

Serum concentrations of INF-γ, TNFα, IL-6, IL-4 and IL-2 were similar between SIBO positive and negative children (*p* > 0.05, Table 3). However, SIBO-positive children showed higher serum concentrations of IL-17 and a lower IL-10 compared to their SIBO-negative counterparts (*p* < 0.05, Table 3).

Logistic regression analysis was performed to determine the influence of variables on SIBO. Two models were used, one analyzing the anti-inflammatory cytokine IL-10 and the other analyzing the inflammatory cytokine IL-6. The models also included other variables such as consumption of ultra-processed foods, gender, BMI and age. In the second model (*p* = 0.01), consumption of ultra-processed foods (*p* = 0.03) and IL-6 levels (*p* = 0.003) were found to contribute to the occurrence of SIBO (Table 4).

**Figure 1:**
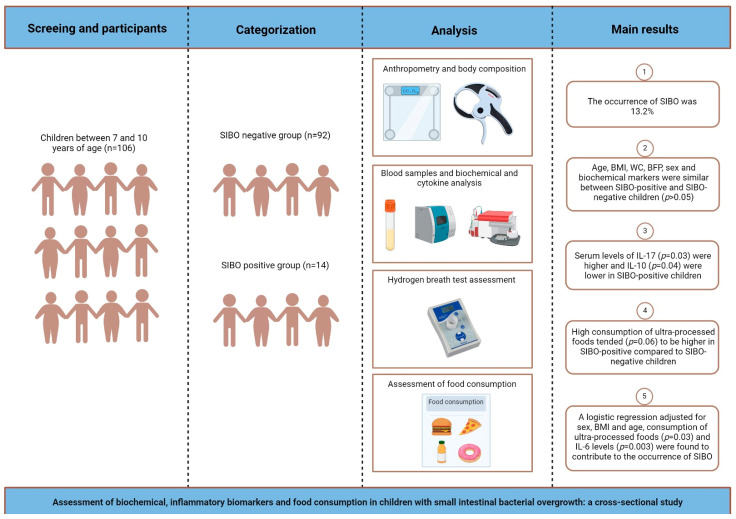
Schematic drawing showing the experimental design and main results obtained in cross-sectional study.

Figure 1 shows the experimental design and the main results.

## 4. Discussion

The study found an occurrence of 13.2% for SIBO in children living in the Northeast of Brazil. In this study, the consumption of ultra-processed foods tended to be higher in SIBO-positive children. In addition, SIBO-positive children had increased levels of IL-17 and decreased levels of IL-10, and logistic regression adjusted for sex, BMI and age showed that consumption of ultra-processed foods and IL-6 levels may be significant predictors of SIBO.

The prevalence of SIBO in the general population can vary from 2.5% to 22%, with age and the occurrence of comorbidities having a positive influence on the risk of SIBO [14]. The data on the epidemiology of SIBO in children are limited due to the relatively small number of studies. The prevalence of SIBO varies according to the clinical context studied, ranging from 8.9% in children using proton pump inhibitors [15] to 91% in children with chronic abdominal pain [16].

Previous studies suggest an association between body composition and the occurrence of SIBO, which can occur in both obesity and underweight. Increased body fat percentage were associated with a greater likelihood of SIBO in children. A study with a small sample of 36 Italian children with excess of adipose tissue found a prevalence of 72.2% of SIBO-positive in children with obesity and overweight [13]. In Brazil, studies carried out in children and adolescents from the south-eastern region found an incidence of 31.5% of SIBO and showed that patients with SIBO had a lower weight-for-age Z score and increased intestinal permeability than those without SIBO [17,18]. In the present study, the BMI was not a predictor for SIBO.

Ultra-processed foods are characterized by being high in energy density, fat, sugar and salt, but low in fiber [19]. Using data from nationally representative dietary surveys conducted between 2004 and 2014, ultra-processed foods accounted for 25% of all calories consumed by children and adolescents in Brazil [20]. About 10 years later, we found an alarming consumption of ultra-processed foods in children, accounting for more than 40% of all calories consumed. We also found a trend towards increased consumption of ultra-processed foods in children with SIBO. A review of meta-analyses showed that greater exposure to ultra-processed foods was associated with the occurrence of several disorders, including gastrointestinal and cardiometabolic disorders [21]. In addition, another meta-analysis of five studies found that the increased risk of SIBO in individuals with obesity did not reach statistical significance, but was significant when only studies from Western countries were included [22], where ultra-processed food consumption is higher [5], which highlights the importance of the present study in discussing the effects of ultra-processed food consumption on SIBO.

High consumption of ultra-processed foods may affect the gut microbiota and its interaction with the gut mucosa, causing structural and functional changes in the gut, leading to local oxidative and inflammatory responses, which may contribute to negative modulation of the gut microbiota and potentially lead to SIBO [23]. Consumption of ultra-processed foods has been identified as one of the factors that may induce stimulation of inflammatory pathways in the gut, which has been identified as one of the causes of SIBO [24,25,26].

There is evidence that lipopolysaccharide production and its increased translocation into the bloodstream can induce chronic systemic inflammation in people with SIBO [27]. This study showed for the first time that serum concentrations of IL-17 were increased while serum levels of IL-10 were decreased in children with SIBO, suggesting a dysfunction of the systemic inflammatory state in these children. The relationship between inflammation and SIBO is a gap that needs further investigation. While one study has shown that the presence of SIBO is associated with elevated concentrations of IL-1β in duodenal fluid [28], others have shown that measures of systemic inflammation do not differ between SIBO-positive and negative children [13,25].

Considering the above, this study provides an innovative perspective on the understanding that ultra-processed food consumption, together with an altered systemic inflammatory parameter, may contribute to the occurrence of SIBO in children. This statement is supported by the results found in this study, which show that the high consumption of ultra-processed foods in combination with high levels of IL-6 in a model adjusted for sex, age and BMI can induce SIBO in these children. A randomized, placebo-controlled trial showed that the administration of a probiotic containing *Saccharomyces boulardii* CNCM I-745 at a dose of 250 mg twice daily for 3 months was effective and safe in eradicating SIBO in decompensated cirrhosis [20]. Whether probiotic therapy is effective in eradicating SIBO in children remains to be investigated. A potential limitation of this study is that symptoms such as abdominal pain, bloating, diarrhea, and constipation were not recorded. Small intestinal cultures could be performed in children who test positive for SIBO on the hydrogen test. In addition, 16S rRNA sequencing could be used for further investigation.

This study identified for the first time an occurrence of 13% of SIBO in children living in the northeastern region of Brazil and showed that consumption of ultra-processed foods and serum levels of IL-6 may influence the occurrence of SIBO in the pediatric population. Although there are currently no guidelines regarding the diagnosis or treatment of SIBO in children, antibiotics remain the first-line approach to SIBO management. However, further studies are needed to know whether dietary interventions, such as low FODMAP (Fermentable Oligosaccharides, Disaccharides, Monosaccharides, and Polyols) or probiotic therapy could be useful in treating SIBO in children.

## Figures and Tables

**Table 1:** Assessment of anthropometric and biochemical variables, age, and sex in children negative and positive for small intestinal bacterial overgrowth (SIBO).

Variables	Negative (*n* = 92)	Positive (*n* = 14)	*p*-Value
Age (years)	9.1 ± 0.9	8.9 ± 0.9	0.28
BMI (kg/m^2^)	22.3 ± 5.3	23.1 ± 5.5	0.57
BMI/A	2.3 (−1.5–5.6)	2.5 (−1.0–4.3)	0.38
WC (cm)	72.9 ± 12.3	75.8 ± 16.2	0.42
BFP (%) †	36.6 (13.7–127.3)	44.7 (21.0–108.7)	0.28
Girls (n(%))	50 (54.3)	8 (57.1)	0.77
Boys (n (%))	42 (45.7)	6 (42.9)	
Albumin (g/dL)	4.5 ± 0.4	4.7 ± 0.3	0.25
AST (U/L)	37.5 ± 9.2	33.3 ± 7.2	0.11
ALT † (U/L)	22.0 (9.0–33.0)	16.5 (7.0–28.0)	0.09
Cholesterol † (mg/dL)	162 (121–278)	165 (144–226)	0.63
LDL-c † (mg/dL)	90 (52–146)	89 (58–137)	0.51
HDL-c(mg/dL)	48.5 ± 11.4	54.1 ± 13.0	0.10
FG (mg/dL)	82 ± 10	79 ± 10	0.31
HbA1c † (%)	5.6 (4.3–9.6)	5.5 (5.2–5.9)	0.31
HOMA-IR †	2.6 (0.4–7.7)	2.8 (0.8–5.1)	0.74
c-RP † (mg/dL)	1.3 (0.1–15.6)	1.2 (0.1–7.1)	0.88

Abbreviations: BMI: body mass index; BFP: body fat percentual; AST: aspartate aminotransferase; ALT: alanine aminotransferase; LDL-c: low-density lipoprotein cholesterol; FG: fasting glucose; HDL-c: high-density lipoprotein cholesterol; HbA1c: Glycated hemoglobin; HOMA-IR: homeostatic model assessment of insulin resistance; c-RP: c-reactive protein. Data are presented as mean and standard deviation or median (minimum–maximum). † non-parametric data.

**Table 2:** Assessment of food consumption in children negative and positive for small intestinal bacterial overgrowth (SIBO).

Variables	Negative (*n* = 92)	Positive (*n* = 14)	*p*-Value
Kcal	2101 ± 439	2015 ± 535	0.51
Unprocessed, (%)	46 ± 10	41 ± 12	0.20
Processed, (%)	17 ± 10	17 ± 9	0.90
Ultra-processed, (%)	37 ± 9	42 ± 9	0.06

Data are presented as mean and standard deviation or median (minimum–maximum).

**Table 3:** Assessment of cytokines in children negative and positive for small intestinal bacterial overgrowth (SIBO).

Variables	Negative (*n* = 92)	Positive (*n* = 14)	*p*-Value
IL-17 †	53.4 (2.3–157.7)	69.5 (5.4–125.7)	0.03
INF-γ †	9.7 (0.9–34.2)	8.3 (0.2–13.1)	0.29
TNFα	4.8 ± 2.7	4.7 ± 3.2	0.85
IL-10 †	5.7 (0.5–30.8)	2.3 (0.6–7.2)	0.04
IL-6 †	10.1 (0.2–95.4)	17.5 (1.6–352.5)	0.08
IL-4 †	4.8 (0.3–28.9)	5.3 (0.4–11.4)	0.09
IL-2 †	7.6 (0.5–39.6)	8.2 (1.9–15.0)	0.80

Abbreviations: IL-17: interleukin 17; INF-γ: interferon-gamma; TNFα: tumor necrosis factor alpha; IL-10: interleukin 10; IL-6: interleukin 6; IL-4: interleukin 4; IL-2: interleukin 2. Data are presented as mean and standard deviation or median (minimum–maximum). † non-parametric data.

**Table 4:** Logistic regression analysis predicting small intestinal bacterial overgrowth (SIBO) with predictors of ultra-processed foods and cytokines serum levels in children.

Variables	Β	SE	t	*p*-Value	*p*-ANOVA	Adjusted R Square
Model 1						
Age	−0.05	0.03	1.51	0.13	0.117	0.037
Sex	0.007	0.06	0.11	0.91		
IL-10	−0.01	0.007	1.82	0.07		
BMI	0.001	0.006	0.27	0.78		
Ultra-processed	0.006	0.003	1.85	0.06		
Model 2						
Age	−0.04	0.03	1.01	0.31	0.01	0.09
Sex	0.001	0.06	0.02	0.98		
IL-6	0.002	0.0008	2.97	0.003		
BMI	0.001	0.006	0.28	0.77		
Ultra-processed	0.007	0.003	2.09	0.03		

Abbreviations: IL-10: interleukin 10; IL-6: interleukin 6; BMI: body mass index.

## Data Availability

The data that support the findings of this study are available from the corresponding author upon reasonable request.
